# 700. LOFI: Preliminary Analysis of Intervention Fidelity of a Cluster Randomized Trial Evaluating Universal Gloving to Reduce *C. difficile* Acquisition within VA Hospitals

**DOI:** 10.1093/ofid/ofad500.762

**Published:** 2023-11-27

**Authors:** Linda McKinley, Cara Ray, Julie Keating, Jonah Dixon, Helene Moriarty, Jiwei Zhao, Charlesnika T Evans, Malini Foliaki, Michael Gelman, Florine Ndakuya-Fitzgerald, Christopher D Pfeiffer, Ahmed Sheeti, Amy Weintrob, Katelyn West, Maeve Williams, Nasia Safdar

**Affiliations:** Wm. S. Middleton Memorial VA Hospital, Madison, Wisconsin; Hines VA Hospital, Hines, Illinois; Madison VA Hospital, Madison, Wisconsin; University of Wisconsin - Madison, Madison, Wisconsin; Villanova University, Philadelphia, Pennsylvania; University of Wisconsin - Madison, Madison, Wisconsin; Northwestern University and VA, Hines, Illinois; Portland VA Hospital, Portland, Oregon; Bronx VA Hospital, Bronx, New York; Milwaukee VA Hospital, Milwaukee, Wisconsin; VA Portland Health Care System, Portland, Oregon; Portland VA Hospital, Portland, Oregon; Washington DC VA Medical Center/ George Washington University, Washington, District of Columbia; Portland VA Hospital, Portland, Oregon; Washington DC VA Hospital, Washington, District of Columbia; University of Wisconsin School of Medicine and Public Health, Madison, Wisconsin

## Abstract

**Background:**

Prevention of *Clostridioides difficile* infection (CDI) is a priority. Infection control measures to prevent CDI target symptomatic (infected) patients. These patients are placed on contact precautions (CP), requiring hand hygiene and use of gloves and gowns. However, asymptomatic (colonized) patients may be a reservoir for cross contamination, and evidence is scarce regarding prevention measures with this population. To address this gap, our study evaluates the effect of universal gloving (UG) for all patient contact to prevent transmission from these patients. However, the gloving intervention involves complex behavioral practices with wide variations in compliance; thus, data on intervention implementation provides critical context. Here we provide a preliminary analysis of intervention fidelity.

**Methods:**

A cluster randomized trial (CRT) is being conducted in ten VA hospital patient units; 5 units randomized to standard of care (CP) and 5 units to the intervention (UG plus CP). A secondary outcome of the study is intervention fidelity (gloving compliance). This analysis includes monthly observation data on hand hygiene (HH) and gloving compliance for UG and HH, gloving, gowning for CP in both groups using a standardized observation tool. Compliance was calculated using the total number of compliant observations divided by the total number of observations.

**Results:**

1012 Observations were conducted between May 2022 and April 2023. HH, gloves and gown compliance for CP did not differ between groups (**Table 1**). Gloving compliance with UG was lower than expected and significantly lower than gloving compliance for CP in the intervention group (**Table 2**). **Table 3** compares HH and gloving between UG (intervention group) and CP (both groups). Gloving compliance was significantly higher with CP than with UG.

Results Tables

**Figure:**
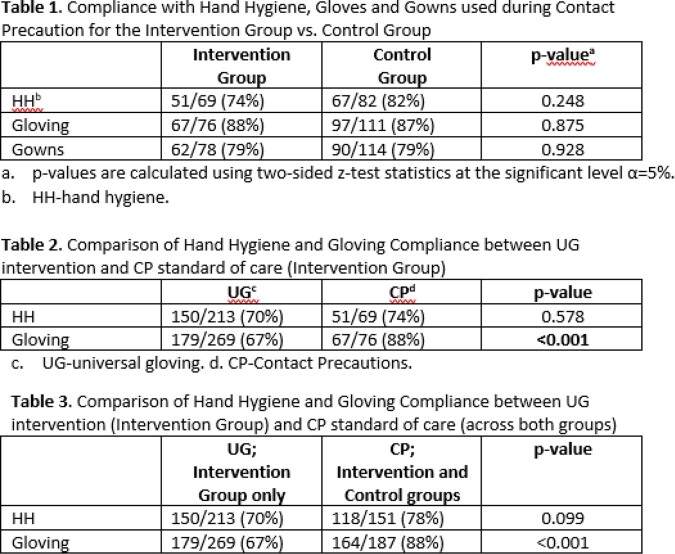

Table 1 - 3

**Conclusion:**

Intervention fidelity is critical to support rigor of the CRT, but our results showed lower than expected compliance for both gloving and HH despite the UG intervention. A pilot interview identified the healthcare worker requirement for practicing both HH and gloving as a barrier. Additional investigation into barriers to gloving and HH compliance within the context of a UG intervention is ongoing.

**Disclosures:**

**All Authors**: No reported disclosures

